# BRD7 Stabilizes P53 via Dephosphorylation of MDM2 to Inhibit Tumor Growth in Breast Cancer Harboring Wild-type P53

**DOI:** 10.7150/jca.67447

**Published:** 2022-02-28

**Authors:** Yanwei Luo, Xinye Wang, Weihong Niu, Yao Zhou, Mengna Li, Jinqi Ma, Jing Yang, Songqing Fan, Zhaoyang Zeng, Wei Xiong, Xiaoling Li, Guiyuan Li, Jidong Xiao, Ming Zhou

**Affiliations:** 1 NHC Key Laboratory of Carcinogenesis, Hunan Cancer Hospital and the Affiliated Cancer Hospital of Xiangya School of Medicine, Central South University, Changsha, Hunan 410013, China.; 2 The Key Laboratory of Carcinogenesis and Cancer Invasion of the Chinese Ministry of Education, Cancer Research Institute, Central South University, Changsha, Hunan 410078, China.; 3 Hunan Key Laboratory of Oncotarget Gene, Hunan Cancer Hospital and the Affiliated Cancer Hospital of Xiangya School of Medicine, Central South University, Changsha, Hunan 410013, China.; 4 Department of Pathology, the Second Xiangya Hospital, Central South University, Changsha, Hunan 410011, China.; 5 Department of Blood Transfusion, the Third Xiangya Hospital, Central South University; Changsha, Hunan 410013, China.; 6 Tongji University Cancer Center, Shanghai Tenth People's Hospital of Tongji University, School of Medicine, School of Life Sciences and Technology, Tongji University, Shanghai 200092, China.; 7 Department of Ultrasonography, the Third Xiangya Hospital, Central South University; Changsha, Hunan 410013, China.

**Keywords:** breast cancer, bromodomain-containing protein 7, p53, ubiquitination, tumor suppressor

## Abstract

Bromodomain-containing protein 7 (BRD7) was found to be down-expressed in nasopharyngeal carcinoma as well as breast cancer and to function as a potential tumor suppressor. BRD7 interacts with p53 and is required for p53-dependent oncogene-induced senescence. However, the mechanism how BRD7 functions as tumor suppressor roles in breast cancer remains unclear. MTT, colony formation assay, cell cycle, cell apoptosis, and tumorigenicity assays were performed to evaluate the biological functions of BRD7 in breast cancer cells *in vitro and in vivo*. Real-time PCR, western blot, luciferase reporter gene assays, and co-immunoprecipitation were used to examine the gene expression, transcription activation and protein-protein interaction. We reported that BRD7 effectively suppressed cell proliferation and tumor growth *in vitro* and* in vivo*. In addition, BRD7 increased p53 protein stability through ubiquitin-dependent proteasome pathway and regulated the expression of p53 downstream target genes by activating its transcriptional activity in breast cancers harboring wild-type p53. Mechanistically, BRD7 decreased phosphorylation and activation of MDM2 via inactivating its upstream kinase AKT depending on the bromodomain of BRD7, therefore BRD7 significantly reduced the amounts of phosphorylated MDM2 binding with p53 eventually decreasing ubiquitination level of p53. Furthermore, silencing the expression of p53 at least partly reversed the inhibition effect of BRD7 on cell proliferation and tumor growth *in vitro* and* in vivo*. Our studies identify that BRD7 stabilizes p53 by inhibiting the phosphorylation of MDM2 via AKT pathway dependent on its bromodomain to function as a tumor suppressor in breast cancer harboring wild-type p53.

## Introduction

Accumulation of deregulation in oncogenes and tumor suppressor genes promotes the development of human cancers. Many genes that are frequently deregulated in breast tumor samples have been identified, such as breast cancer 1 (BRCA1), tumor protein 53 (TP53), ataxia telangiectasia mutated (ATM) and bromodomain-containing protein 7 (BRD7) 1. BRD7 is a subunit of the SWI/SNF chromatin remodeling complex, which recruits BRCA1 and Oct-1 protein to the ER-α promoter, and then regulates the ER-α transcription activity and expression 2, 3. Our previous studies showed that BRD7 acted as a tumor suppressor in nasopharyngeal carcinoma (NPC) by regulating Wnt/β-catenin, ras/MEK/ERK, Rb/E2F and PTEN/AKT pathways 4-7, and BRD7 interacted with other members of bromodomain-containing protein family members, such as BRD2 and BRD3, to trigger apoptosis and induce cell cycle arrest at G1-S phase 8-10. As a transcription factor, BRD7 binds to histone H3 through its bromodomain to promote acetylation, and results in activation of E2F3 promoter activity 11. On the other hand, the interaction of BRD7 with protein arginine methyltransferase 5 (PRMT5) is involved in transcriptional repression of their target genes 12. Moreover, our previous results show that BRD7 functions as a potential tumor suppressor in breast cancer which could inhibit cell proliferation, migration, tumor growth and invasion as well as promote cell apoptosis and drug-sensitivity to paclitaxel 13, 14.

BRD7 has been identified as a p53 cofactor, and is required for efficient p53-mediated transcription in a subset of target genes by recruiting p300 to target gene promoters 15, 16. p53 can regulate the expression of many target genes including tumor suppressors or oncogenes to induce cell cycle arrest, apoptosis, and senescence, thereby preventing cells from malignant transformation and tumorigenesis 17, 18. P53 is a highly unstable protein which is regulated by ubiquitin-proteasome pathway. The function of p53 is frequently lost in cancers, which is mainly induced by overexpression of negative regulators MDM2 19. MDM2 is a ubiquitin E3 ligase that is highly expressed in many human tumors and mediates the ubiquitination of p53, and results in degradation of p53 20, 21. Therefore, restoration of wild-type p53 function by abrogating the MDM2-p53 interaction and inhibiting the enzymatic activity of the p53 ubiquitination pathway has become an attractive therapeutic strategy 22, 23.

In this study, we demonstrated the ability of BRD7 to suppress cell proliferation and tumor growth *in vitro* and *in vivo*. In addition, BRD7 increased p53 protein stability through ubiquitin-dependent proteasome pathway as well as changed the expression of downstream target genes by activating p53 transcriptional activity in breast cancers harboring wild-type p53. Mechanistically, BRD7 decreased activation of MDM2 thus reducing the amount of activated MDM2 binding with p53 and eventually increasing p53 stability. Furthermore, silencing the expression of p53 at least partly attenuated the tumor suppressive effect of BRD7 on cell proliferation and tumor growth. Taken together, our study identifies that BRD7 stabilizes p53 by regulating the phosphorylation of MDM2 via AKT pathway dependent on its bromodomain (BRD) to function as a tumor suppressor in breast cancer harboring wild-type p53.

## Materials and Methods

### Cell culture

The human breast cancer cell lines with wild-type p53, MCF-7 and ZR-75-1, as well as the cell line with mutant-type p53, MDA-MB-231, were purchased from ATCC (The Global Bioresource Center). These cells were cultured in DMEM (Invitrogen Life Technologies, Carlsbad, CA, USA) supplemented with 10% fetal bovine serum (FBS) (Gibco-BRL, Invitrogen, Paisley, UK) in a humidified incubator containing 5% CO2 at 37 °C.

### Cell treatment

To overexpress wild-type BRD7 (WT BRD7) or the mutant-type BRD7 with bromodomain deletion (BRD7∆brd) in breast cancer cell lines, ZR-75-1 and MCF-7 cells were transfected with the plasmid pIRES2-BRD7 or pIRES2-BRD7∆brd for 48 h using Lipofectamine 3000 (Life technologies, Grand Island, NY) in accordance with the manufacturer's instruction. The cells transfected with empty plasmid pIRES2 were used as a vector control. In order to increase the protein concentration in the cells, the transfected MCF-7 cells were treated with proteasome inhibitor MG-132 (10 μM dilution in DMSO) (Selleck, Houston, TX, USA) for different time (2, 4, 6, 8, 16 h). To test the half-life of p53, the transfected MCF-7 cells were treated with cycloheximide (CHX, 20 μg/ml dilution in ethyl alcohol) (Sigma-Aldrich) for different time (1, 30, 60, 120, 240 min). To knock down the expression of p53, MCF-7 cells were simultaneously transfected with three small interfering RNA (siRNA) sequences for 48 h (#1: 5'- GACUCCAGUGGUAAUCUAC dTdT3'; #2, 5'-GAAAUUUGCGUGUGGAGUAdTdT-3'; #3, 5'-CUGCCCUCAACAAGAUGU UdTdT-3') by using Lipofectamine 3000 (Life technologies, Grand Island, NY) in accordance with the manufacture's instruction. A siRNA scramble sequence was used as negative control (cat no: Q000007157-1-B, RiboBio Co., Ltd, Guangzhou, China).

### Immunohistochemical staining

The paraffin-embedded slides were routinely deparaffinized and hydrated. The slides then were retrieved in citric acid buffer (pH6.0) heated by microwave for 20 min. The slides then were treated with 3% H_2_O_2_ for 15 min and washed by tris-buffered saline containing 0.1% Tween 20 (TBST) three times each for 5 min. After 60 min incubation in normal goat serum (Boster, Wuhan, China), slides were incubated with primary antibody (polyclone rabbit anti-BRD7 (1:500 dilution) from Proteintech, Wuhan, China; polyclonal rabbit anti-p53 (1:500 dilution), anti-phosphorylated MDM2 at ser166 (pMDM2, 1:1000 dilution), anti-c-PARP (1:200 dilution) and anti-p21 (1:200 dilution) from Cell Signaling Technology, Danvers, MA; polyclonal rabbit anti-Ki67 (1:200 dilution) from Bioworld Technology, Inc. St. Louis, Minnesota, USA) overnight at 4 °C. The slides were washed three times with TBST (each for 15 min) and then incubated with a secondary goat anti-rabbit antibody (Maixin, Fujian, China) for 60 min at 37 °C. Then the slides were visualized with 3,3'-diaminobenzidine (DAB) (Zhongshan Gold bridge, Beijing, China) for 5 min and counterstained with haematoxylin for 45 seconds. The slides were mounted and photographed with Olympus BX51 microscope (Olympus, Japan).

### Evaluation of staining

To semi-quantify the expression of BRD7, p53, pMDM2, p21, c-PARP and Ki67 in the tumor tissues from nude mice, ImageJ (NIH) was used to access the integral optical density (IOD) of immunoreactivity, and the detail evaluation of staining was referred to the previous publication^24^, ^25^. Data were acquired from eight sections/animal and averaged to produce a single value per subject.

### Western blot

Protein was extracted from indicated cells by using RIPA lysis buffer. The protein concentrations were determined by using BCA Protein Assay kit (Thermo Fisher Scientific, Rockford, IL, USA). Total of 60 μg protein was added and separated on a 10% SDS-PAGE gel and transferred onto a polyvinylidene difluoride (PVDF) membrane (Millipore, Billerica, MA, USA). Following incubation in 5% nonfat milk for 2 h, the membrane was then incubated with primary antibody (polyclonal rabbit anti-BRD7 (1:500 dilution), anti-Bcl-2 (1:500 dilution), anti-β-Tubulin (1:500 dilution), and anti-Bax (1:500 dilution) from Proteintech, Wuhan, China; polyclone rabbit anti-p53 (1:500 dilution), anti-pMDM2 (1:1000 dilution), anti-total-PARP (1:1000 dilution), anti-c-PARP (1:500 dilution), anti-AKT (1:1000 dilution), anti-pAKT (1:1000 dilution), anti-Bak (1:1000 dilution), anti-Flag (1:1000 dilution), and anti-p21 (1:1000 dilution) from Cell Signaling Technology, Danvers, MA; monoclonal rabbit anti-total-MDM2 (1:1000 dilution) from Abcam, Cambridge, UK, and polyclonal rabbit anti-cyclin D1 (1:1000 dilution) and monoclonal mouse anti-GAPDH (1:3000 dilution) from Santa Cruz, Dallas, Texas, USA overnight at 4 °C. After washing with TBST 3 times for 8 min each time, the membrane was incubated with the appropriate secondary antibody for 1 hour at 37 °C. The membranes were then washed with TBST and visualized the bands by using an ECL kit (Millipore). Image-J (NIH) was used to analyze the relative protein expression which was normalized to GAPDH.

### Co-immunoprecipitation

The indicated cells were added 200μl IP lysis buffer (Boster) and slowly shaking for 30 min on ice. The lysis was centrifuged at 13000 g for 15 min and the supernatant were collected to incubate with 10 μg of antibody (BRD7, p53 or MDM2) overnight at 4 °C. The next day, the supernatant was incubated with protein A/G agarose beads (Proteintech) for 5 h, and then centrifuged at 3000 g for 5 min at 4 °C. The precipitates were collected and detected by western blot with the appropriate antibodies.

### Real-time quantitive polymerase chain reaction (qPCR)

Total RNA was extracted by using Trizol Reagent (Life Technologies). Reverse Transcription Kit (Thermo Fisher scientific) was used to convert RNA into cDNA according to the manufacturer's instruction. Real-time qPCR was then performed by using EvaGreen qPCR MasterMix (Applied Biological Materials, Richmond, Canada) on CFX connect Real-Time system (Bio-Rad, Hercules, CA, USA) according to the manufacturer's instructions. The primers were used as followings: BRD7, forward: AACGACGTTGGGACTTCTCC, reverse: TGCTCCATTTCTTTTGCTGTGT; p53, forward: ACCTATGGAAACTACTTCCTGAAA, reverse: ACCATCGCTATCTGAGCAGC; bcl-2, forward: TTTGTGGAACTGTACGGCCC, reverse: ACAGCCTGCAGCTTTGTTTC; Bax, forward: GGCCCTTTTGCTTCAGGGTT, reverse: AGCTGCCACTCGGAAAAAGA; Bak, forward: AGGATCCCGGCAGGCTGAT, reverse: AACGTAGCTGCGGAAAACCT; p21, forward: GCTGCCGAAGTCAGTTCCTT, reverse: ATCTGTCATGCTGGTCTGCC; Cyclin D1, forward: ACGGACTACAGGGGAGTTTTG, reverse: GAAATCGTGCGGGGTCATTG; GAPDH, forward: TGGGCAGCCGTTAGGAAAG, reverse: GACTCCACGACGTACTCAGC. The qPCR steps were 95 °C for 10 min, and 40 cycles of denaturation at 95 °C for 15 sec and anneal/extend step at 60 °C for 60 sec. The target genes expression was normalized to GAPDH. The relative expression was analyzed by the 2^-ΔΔCt^ method.

### MTT assay

Cell proliferation was measured by MTT assay. After indicated treatment, the cells were resuspended and counted. One thousand cells were seeded into a 96-well plate and continuously cultured for 0, 24, 48 and 72 h. At the time point, 200 μl MTT (Sigma-Aldrich, St. Louis, MO, USA) at a 0.5 mg/ml final concentration was added into each well and incubated for 4 h at 37 °C. And then 150 μl of DMSO (Sigma-Aldrich) was added to each well and incubated for 15 min at room temperature. Finally, the optical density (OD) at 490 nm was detected by using a PARADIGM Detection Platform (Beckman, Brea, CA). This assay was repeated 3 independent times.

### Colony formation assay

Colony formation assay was conducted to examine the colony formation capacity of breast cancer cells, MCF-7 cells *in vitro*. Two hundred cells in each group were added to each well of a 6-well plate. After cultured for 14 d, cells were gently washed by PBS and stained with 0.1% crystal violet. The stained colonies were counted.

### Cell apoptosis assay

For cell apoptosis assays, Annexin V-PE/7AAD (7-amino-actinomycin D) Apoptosis Detection Kit (KeyGEN Biotech, Nanjing, China) was used. After indicated treatment, the cells were collected and washed with ice PBS twice and then resuspended in 500 μl 1 × binding buffer. 5 μl Annexin V-PE and 5 μl 7AAD were added, mixed well, and incubated for 15 min protected from light. Cells were analyzed by using BD LSRFortessa (Becton, Dickinson and Company, Franklin Lakes, New Jersey, USA). This assay was repeated 3 independent times.

### Cell cycle analysis

Cells from each group were trypsinized and washed with cold PBS. The cells were incubated in 75% alcohol at 4 °C overnight. Then, the cells were washed and treated with RNase A (TaKaRa, Japan) for 15 min at 37 °C and stained with prodiumiodide (PI) (KeyGEN Biotech) for 15 min protected from light. The cell cycle was analyzed by using BD LSRFortessa (Becton, Dickinson and Company). This assay was repeated 3 independent times.

### Luciferase reporter gene assays

The p53 transcription region was ligated into the pGL3-echancer vector (Promega) to generate recombinant luciferase reporter gene (Luc-p53). The Luc-p53 plasmids were kindly provided by prof. Zeng Zhaoyang. The MCF-7 cells were transfected with the Luc-p53 alone, or co-transfected with BRD7 expressed plasmid or vector control. And the cells transfected with empty pGL3-echancer vector were used as control. At 48 h after transfection, cells were collected and lysed. 100 μl of the supernatants were used to detect luciferase activities by using luciferase reporter gene assay kit (Promega) on PARADIGM Detection Platform (Beckman, Brea, CA).

### *In vivo* tumorigenicity assays

Xenograft tumorigenicity assays were approved by the Institutional Animal Care and Use Committee of Central South University. Four-week-old female athymic nude mice were purchased from Hunan SJA LABORATORY ANIMAL CO., LTD, housed at Department of laboratory Animals of Central South University. All animals were allowed access to standard chow diet and water ad libitum and were housed in a pathogen free barrier facility with a 12 L:12D cycle. The mice were divided into four groups, and each group contains six mice. Before injection of MCF7 cells, the mice were injected intramuscularly with 0.2 mg of 17-β-estradiol in 10 μl of ethyl alcohol (Tokyo Chemical Industry, Tokyo, Japan) every two days for 6 days. After indicated treatment, 5×10^6^ MCF-7 cells in 100 μl of serum-free medium were injected subcutaneously into subaxillary of right forelimb. Mice were monitored and tumor volumes were measured every two days for 24 days after injection. The tumor volume was equivalent to 1/2 (length×width^2^). Tumor weight was also recorded when mice executed by CO_2_ asphyxiation.

### Statistical analysis

GraphPad Prism 6 software (Graphpad Software, Inc., La Jolla, CA, USA) and SPSS 16.0 were used to perform statistical analysis. The data are presented as the mean ± standard deviation. Student's t-test and ANOVA were used depending on experimental conditions. A value of P<0.05 was statistically significant.

## Results

### Overexpression of BRD7 inhibits cell proliferation, arrests cell cycle at G1/S phase and induces apoptosis

**Figure 1:**
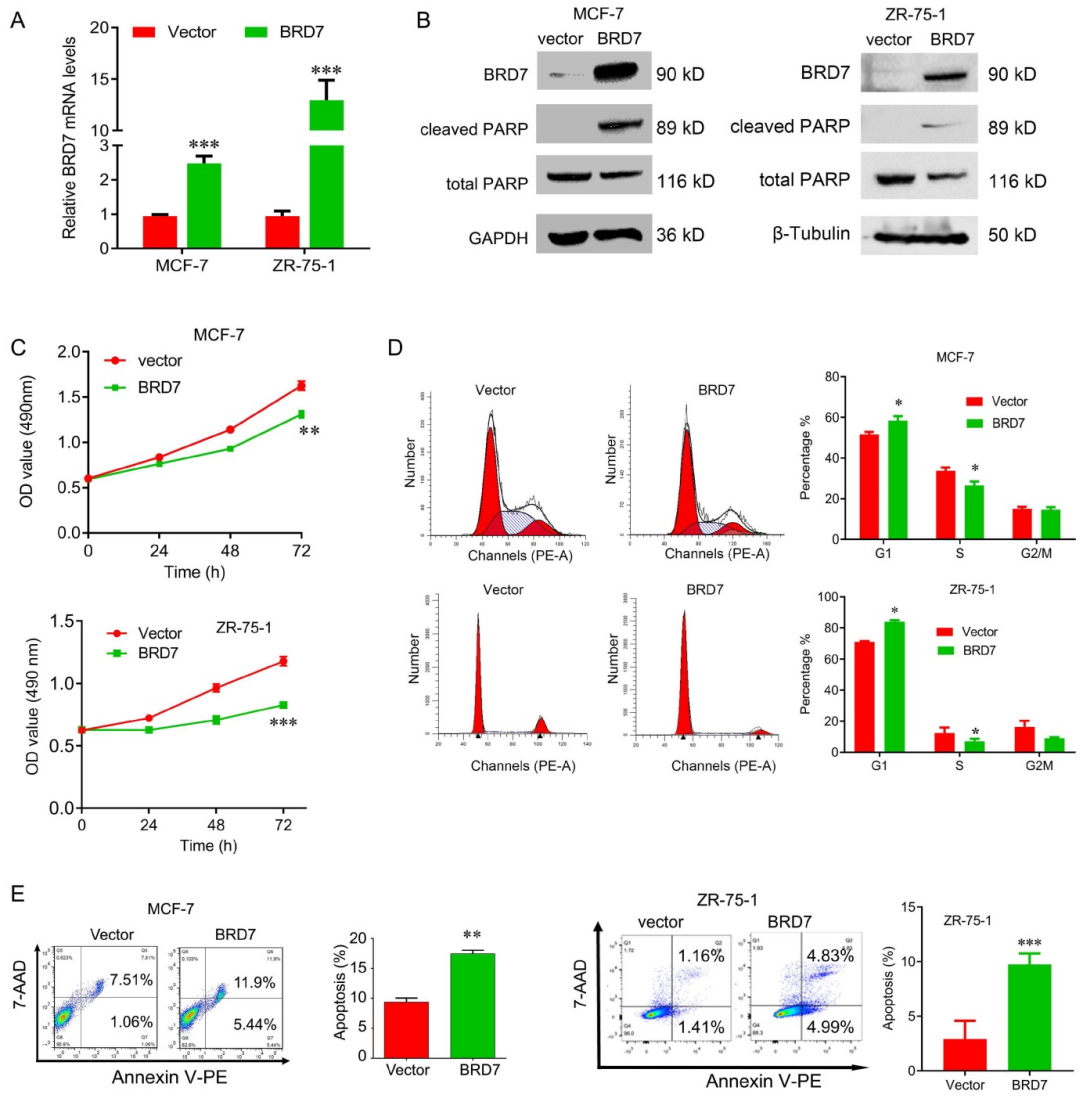
** BRD7 inhibits cell proliferation and induces cell apoptosis in breast cancer cells. (A)** Expression levels of BRD7 in MCF-7 and ZR-75-1 cells transfected with empty vector control or BRD7 expressed plasmid. **(B)** Western blot analysis for BRD7, cleaved PARP, and total PARP in MCF-7 and ZR-75-1 cells transfected with plasmid vector or BRD7 expressed plasmid. **(C)** MTT assay in MCF-7 (upper) and ZR-75-1 (lower) cells transfected with plasmid vector or BRD7 expressed plasmid. **(D)** Cell cycle analysis in MCF-7 and ZR-75-1 cells transfected with plasmid vector or BRD7 expressed plasmid. **(E)** Flow cytometric analysis for cell apoptosis in MCF-7 and ZR-75-1 cells transfected with plasmid vector or BRD7 expressed plasmid. Data are expressed as mean ± s.d. *p < 0.05, **p < 0.01, ***p < 0.001.

To explore the tumor suppressor roles of BRD7 in breast cancer harboring wild-type p53, we transfected the BRD7-expressed plasmids into ZR-75-1 and MCF-7 cells to construct the stable cell lines with ectopic expression of BRD7, and the overexpression efficiency of BRD7 was confirmed by qRT-PCR and western blotting (Figure 1A, B). We then performed a battery of experiments *in vitro* and found that overexpression of BRD7 significantly inhibited cell proliferation and cell cycle progression from G1 to S phase, and increased the apoptosis rate in MCF-7 and ZR-75-1 cell lines (Figure 1C, D, E). We also detected the two apoptosis markers, cleaved PARP and total PARP, and found that ectopic expression of BRD7 significantly changed the expression of these two markers (Figure 1B). Thus, these results reveal that BRD7 inhibits cell proliferation and cell cycle progression from G1/S phase, and induces apoptosis in breast cancer cells harboring wild-type p53.

### BRD7 activates the p53 transcriptional activity

**Figure 2:**
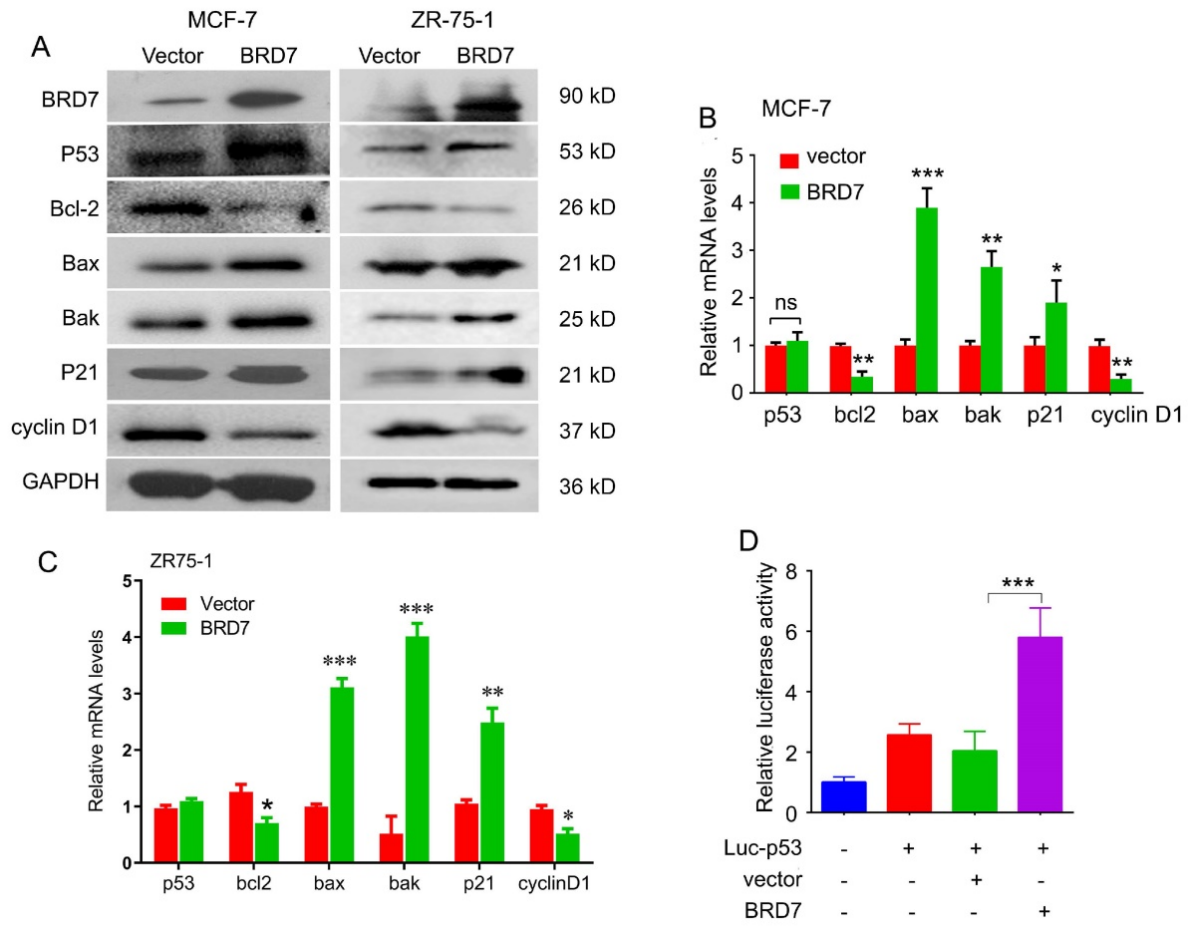
** BRD7 regulates p53 protein expression and its downstream signaling. (A)** Western blot analysis for p53 and its target genes, Bcl-2, Bax, Bak, p21 and cyclin D1 in MCF-7 and ZR-75-1 cells transfected with plasmid vector or BRD7 expressed plasmid. **(B, C)** Relative mRNA levels of p53 and its target genes, Bcl-2, Bax and Bak, in MCF-7 and ZR-75-1 cells transfected with plasmid vector or BRD7 expressed plasmid measured by real-time PCR. **(D)** BRD7 upregulated the p53 transcriptional activity in MCF-7 cells. Data are expressed as mean ± s.d. *p < 0.05, **p < 0.01, ***p < 0.001.

BRD7 has been identified as a co-factor with p53. To determine whether BRD7 can regulate p53 expression and its downstream molecules, we performed western blot and qPCR to detect the expression of p53 and its target genes in breast cancer cell lines harboring either wild-type p53 or mutant p53. As a result, ectopic expression of BRD7 induced the expression of p53 at protein level both in MCF-7 and ZR-75-1 cells harboring endogenous wild-type p53, and subsequently regulated the expression of p53 target genes, including Bcl-2, Bax, Bak, p21 and cyclin D1 protein level (Figure 2A). We also found overexpression of BRD7 could regulate the expression of p53 target genes at mRNA level, but could not change the mRNA expression of p53 in these breast cancer cells with endogenous wild-type p53 (Figure 2B, C). However, when we performed the same experiments on MDA-MB-231 cells harboring mutant p53, we observed that BRD7 failed to changing the expression of endogenous mutant p53 as well as its target genes both at the level of mRNA and protein (Figure S1). These results indicated that BRD7 could increase the protein expression of endogenous wild-type p53 at post-transcription level. Since BRD7 could transcriptionally change the expression of p53 target genes, we thus investigated the effect of BRD7 on p53 transcriptional activity. Luciferase reporter vector containing the p53 transcriptional targeting region (Luc-p53) was used, and the results show that overexpression of BRD7 greatly increased p53 transcription activity on its target gene in MCF-7 cells (Figure 2D). These results suggest that BRD7 can increase p53 protein levels and its downstream target genes expression by activating p53 transcriptional activity.

### BRD7 decreases ubiquitination of p53 protein

**Figure 3:**
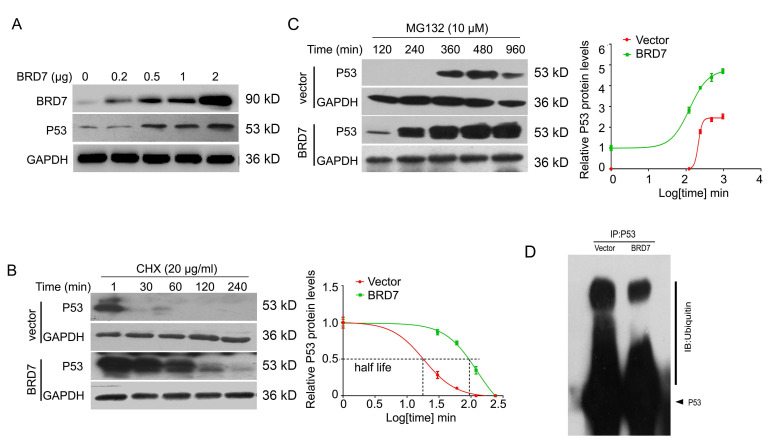
** BRD7 stabilizes p53 protein expression. (A)** Western blot analysis for BRD7 and p53 in MCF-7 cells transfected with increased amount of BRD7 expressed plasmid. **(B)** Western blot analysis for BRD7 and p53 in MCF-7 cells transfected with vector plasmid or BRD7 expressed plasmid, and then treated with cycloheximide (CHX) for 1, 30, 60, 120 and 240 min (left), and quantification of the bands of western blot with Log transformed time (right). **(C)** Western blot analysis for BRD7 and p53 in MCF-7 cells transfected with plasmid vector or BRD7 expressed plasmid, and then treated with MG132 for 120, 240, 360, 480 and 960 min (left), and quantification of the bands of western blot with Log transformed time (right). **(D)** Overexpression of BRD7 inhibited the ubiquitination of p53 in MCF-7 cells. Cells were transfected with plasmid vector or BRD7 expressed plasmid. Cell lysates were immunoprecipitated with the p53 antibody and then immunoblotted with the ubiquitin antibody.

Since BRD7 could increase the protein expression of p53 at post-transcriptional level in breast cancer cells harboring wild-type p53, we then aim to investigate whether BRD7 could stabilize p53 protein to protect it from degradation by ubiquitination. To test this hypothesis, we transfected BRD7 expressing vector with increasing dose into MCF-7 cells, and the results showed that BRD7 overexpression upregulated the endogenous p53 protein levels in a dose dependent manner in MCF-7 cells (Figure 3A). To determine whether BRD7 increases the p53 protein half-life, MCF-7 cells transfected with BRD7 expressing vector or empty vector were treated with protein synthesis inhibitor cycloheximide (CHX) for different time periods, respectively. As a result, ectopic BRD7 clearly increased the half-life of p53 compared with cells transfected with control vector (124 min versus 18 min, Figure 3B), which suggests that BRD7 stabilizes p53 protein to protect it from degradation. To clarify the mechanism of BRD7 stabilizing p53 protein, we next treated MCF-7 cells that transfected with BRD7 expressing vector or empty vector with proteasome inhibitor MG132, and found that BRD7 overexpression accumulated more p53 protein (Figure 3C). We further investigated whether BRD7 stabilized p53 protein through ubiquitination. The ectopic WT BRD7 clearly inhibited p53 ubiquitination in MCF-7 cells (Figures 3D).

### BRD7 decreases the binding of p53 with phosphorylated MDM2 depending on bromodomain

**Figure 4:**
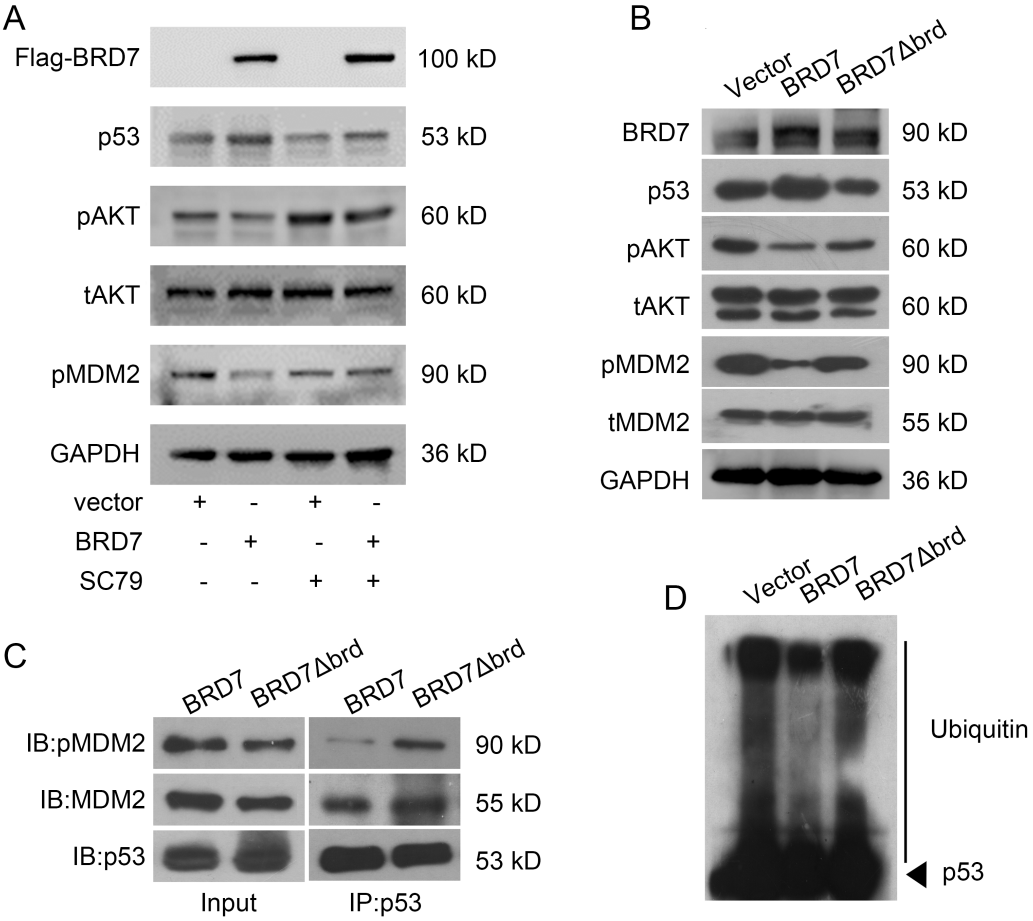
** Bromodomain of BRD7 is required for interaction of p53 and MDM2 as well as stabilization of p53. (A)** Western blot analysis for BRD7, p53, phosphorylated AKT, total AKT (t-AKT), and phosphorylated MDM2 (pMDM2) in MCF-7 cells transfected with empty vector or WT BRD7 expressed plasmid in the existence of AKT activator SC79. **(B)** Western blot analysis for BRD7, p53, phosphorylated AKT, total AKT (t-AKT), phosphorylated MDM2 (pMDM2) and total MDM2 (tMDM2) in MCF-7 cells transfected with empty vector or WT BRD7, or BRD7 ∆brd expressed plasmids. brd, bromodomain. **(C)** The interaction between p53 and pMDM2, or tMDM2 in MCF-7 cells transfected with empty vector or WT BRD7, or BRD7∆brd expressed plasmid. IP, immunoprecipitation; IB, immunoblotting. **(D)** Overexpression of WT BRD7 but not BRD7∆brd inhibited the ubiquitination of p53 in MCF-7 cells. Cells were transfected with plasmid vector or WT BRD7, or BRD7∆brd expressed plasmid. Cell lysates were immunoprecipitated with the p53 antibody and then immunoblotted with the ubiquitin antibody.

As phosphorylation of MDM2 is the activated form for p53 ubiquitylation, and AKT is the main upstream factor regulating phosphorylation of MDM2, while BRD7 is known as a p85α-interacting protein that negatively regulates AKT signaling 26, we thus test the effect of BRD7 on AKT phosphorylation. As a result, overexpression of BRD7 could decrease MDM2 phosphorylation as well as the phosphorylation level of AKT, while restoration of AKT phosphorylation with AKT activator SC79, the phosphorylation level of MDM2 was accordingly recovered (Figure 4A). These results suggest BRD7 decreases MDM2 phosphorylation mainly due to the inhibition of AKT activation by BRD7.

Since the bromodomain of BRD7 is critical for its tumor suppressor activity 10, 11, we then tested whether the bromodomain of BRD7 was required for the inhibition of MDM2 phosphorylation. Our results showed that BRD7 could decrease the phosphorylation levels of AKT and MDM2 as the previous results, while the mutant BRD7 with bromodomain deletion (BRD7∆brd) could not change the levels of phosphorylated AKT and MDM2 as well as the protein level of p53 (Figures 4B). As p53 ubiquitylation depends on the binding of p53 with activated MDM2, we thus detected the effect of BRD7 and BRD7∆brd on the binding of p53 with activated MDM2 as well as the ubiquitylation level of p53. As expected, the existence of wild-type BRD7, but not BRD7∆brd, significantly reduced the amounts of phosphorylated MDM2 binding on p53 (Figure 4C), and BRD7∆brd overexpression disabled to decrease the levels of p53 ubiquitination (Figure 4D). These results demonstrated that BRD7 decreased the binding of phosphorylated MDM2 on p53 thus decreasing p53 ubiquitylation and increasing p53 protein stability dependence on bromodomain of BRD7.

### The tumor suppressor role of BRD7 is partly dependent on p53 and its bromodomain

**Figure 5:**
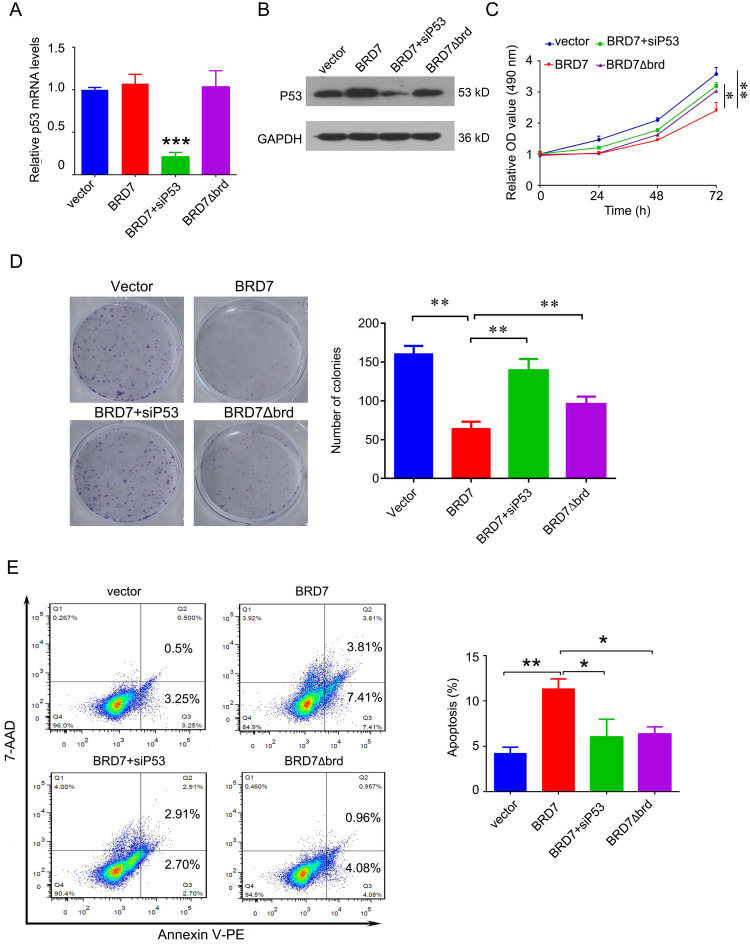
** The tumor suppressor role of BRD7 is partly dependent on p53 and its bromodomain. (A)** Relative mRNA levels of p53 in MCF-7 cells transfected with blank vector, or BRD7 expressed plasmid with or without siRNA p53 plasmid, or BRD7∆brd expressed plasmid alone measured by real-time PCR. **(B)** Western blot analysis for p53 in MCF-7 cells transfected with blank vector or BRD7 expressed plasmid with or without siRNA p53 plasmid, or BRD7∆brd expressed plasmid alone. **(C)** MTT assay in MCF-7 cells transfected with indicated plasmids. **(D)** Represented images of colonies on 6-well plate for MCF-7 cells, and quantification of number of colonies per well. **(E)** Flow cytometric analysis for cell apoptosis in MCF-7 cells transfected with indicated plasmids. Data are expressed as mean ± s.d. *p<0.05, **p < 0.01, ***p < 0.001.

To investigate whether the tumor suppressor role of BRD7 is dependent on p53 and its bromodomain, we transfected the vectors expressing wild-type BRD7 alone, or the vectors expressing BRD7∆brd alone, or co-transfected the wild-type BRD7 vector and p53 siRNA pools into MCF-7 cells. The transfection efficiency was confirmed by qPCR and western blotting. As a result, siRNA-p53 transfection significantly decreased the expression of p53 after BRD7 overexpression, while BRD7∆brd did not alter the expression of p53 compared with vector control (Figure 5A, B). In the functional experiments, we found that downregulation of p53 partly attenuated the effects of BRD7 on cell proliferation, colony formation, and apoptosis (Figure 5C, D, E). In addition, BRD7 with bromodomain deletion mutant has only weaker effects on cell proliferation, colony formation, and apoptosis than wild-type BRD7 (Figure 5C, D, E). These results suggest that the tumor suppressor role of BRD7 is at least partly dependent on p53 and its bromodomain in breast cancer with wild-type p53.

### P53 can rescue the tumor suppressive effect of BRD7 on tumor growth *in vivo*

**Figure 6:**
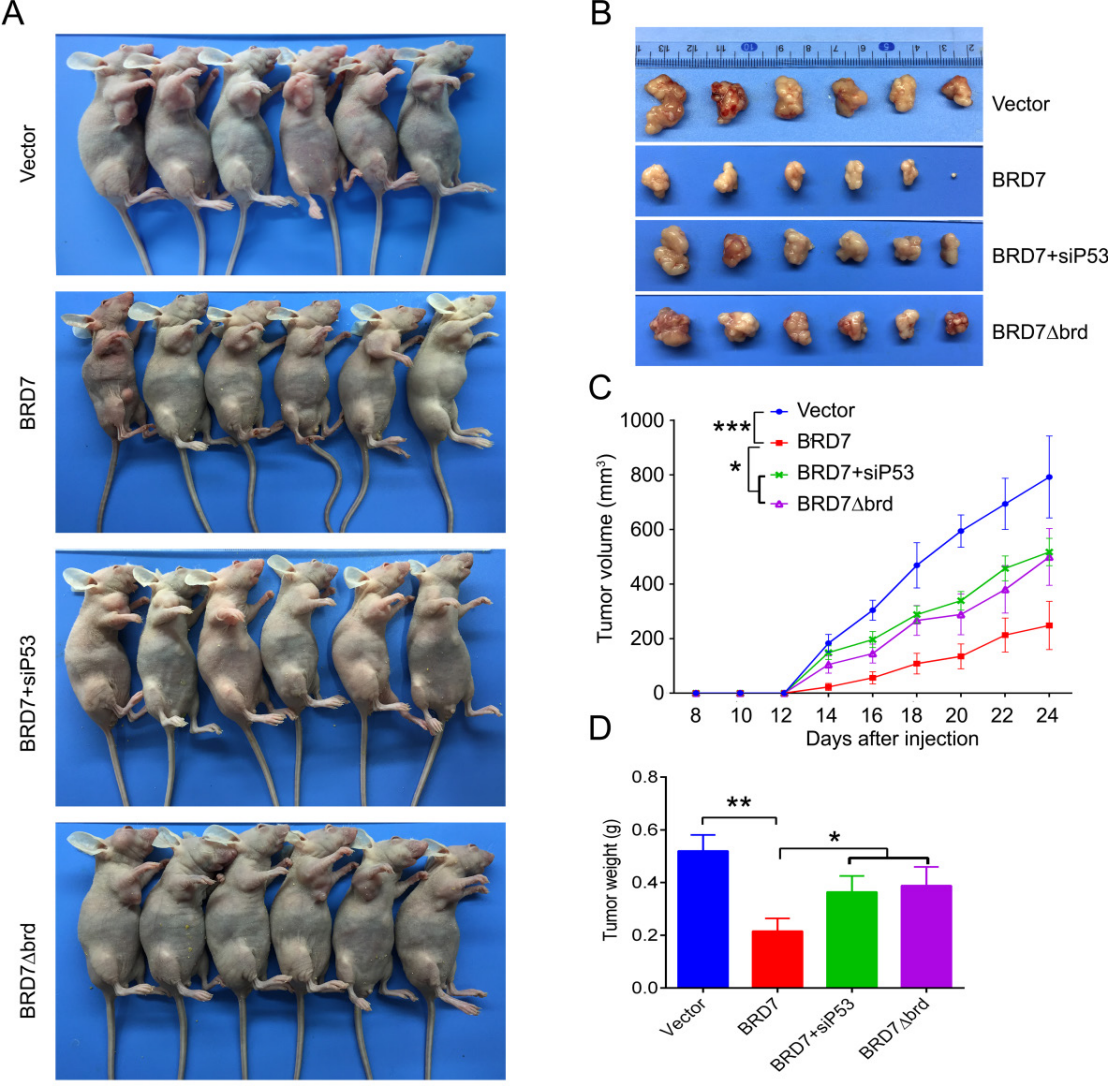
** P53 can rescue the tumor suppressive effect of BRD7 on tumor growth *in vivo*. (A)** The MCF-7 cells that transfected with vector plasmid, or BRD7 expressed plasmid with or without siRNA p53 plasmid, or BRD7∆brd expressed plasmid alone were subcutaneously injected into nude mice. **(B)** The tumors were obtained from the mice. **(C)** The tumor volume was monitored every two days. **(D)** Measurement of the tumor weight across groups. *p<0.05, **p < 0.01, ***p < 0.001.

**Figure 7:**
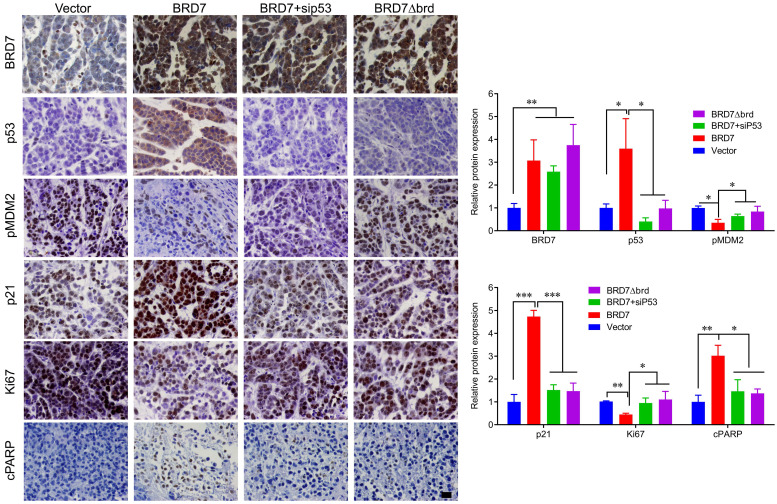
** IHC detection of tumor samples from xenografted tumor in mice.** Representative images of immunohistochemistry staining for BRD7, p53, phosphorylated MDM2 at ser166 (pMDM2) and p21, and the markers of proliferation and apoptosis, Ki67 and c-PARP in tumor tissues, and quantification of integral optical density (IOD). Data are expressed as mean ± s.d. *p<0.05, **p < 0.01, ***p < 0.001.

We have demonstrated that BRD7 could suppress cell proliferation and induce apoptosis by stabilizing p53 and dependent on it bromodomain *in vitro* in breast cancer cells harboring wild-type p53. We next tested whether BRD7 could suppress tumor growth of breast cancer cells xenografted tumors through stabilization of p53 *in vivo*. Nude mice were subcutaneously injected with MCF7 cells that transfected with WT BRD7-overexpressing vector alone, or BRD7∆brd-expressing vector alone, or co-transfected the wild-type BRD7 and p53 siRNA, respectively, and the empty vector group was used as a control. Tumor formation was monitored every two days. As expected, the tumor volume and tumor weight were significantly decreased in mice injected with wild-type BRD7-overexpressing plasmid compared with the vector-control mice, while restoration of p53 or deletion of bromodomain partly attenuated the inhibitory effects of BRD7 on tumor growth (Figure 6A-D). Moreover, we detected the expression of BRD7, p53, phosphorylated MDM2 and p21 as well as the markers of proliferation and apoptosis, Ki67 and c-PARP, in tumor tissues by IHC. Consistent with the results of experiments *in vitro*, wild-type BRD7 but not BRD7∆brd significantly increased the levels of p53 and p21 protein and decreased the levels of phosphorylated MDM2 (Figure 7). The expression of Ki67 was clearly reduced, while the expression of c-PARP was increased in BRD7-overexpression group. However, their expression was rescued after restoration of p53 and deletion of bromodomain of BRD7 (Figure 7). These data further support that BRD7 is partly dependent on p53 and its bromodomain to function as tumor suppressor in breast cancer harboring wild-type p53.

## Discussion

BRD7 is initially identified as a tumor suppressor in nasopharyngeal carcinoma (NPC) in our lab 6. Subsequent studies in our team and other teams also find that BRD7 functions as a tumor suppressor in non-small cell lung cancer (NSCLC), osteosarcoma, endometrial carcinoma, and prostate cancer 27-30. Mechanistically, BRD7 exhibits the inhibitory function in malignant cells through regulation of β-catenin, Ras/MEK/ERK, Rb/E2F, and PI3K/AKT pathways 4, 5. Herein, we demonstrated that overexpression of BRD7 in breast cancer cells harboring wild type p53 exhibited the tumor suppressive effects assessed by cell viability, cell cycle, apoptosis, and xenograft tumor model. Our previous study has found that BRD7 sensitizes breast cancer cells MDA-MB-231 harboring mutant type p53 to paclitaxel through activating Bak 13. When the DNA is damaged by external factors and is difficult to repair, wild-type p53 quickly induces cell apoptosis and prevents the production of potentially cancerous cells, thereby effectively exerting a tumor suppressor effect. Hu et al found that the degradation of BRD7 resulted in cancer cell resistance to DNA-damaging agents 31. However, mutant p53 acts as a proto-oncogene, which stably exists in tumor cells and accumulates in the nucleus of tumor cells, ultimately leading to the occurrence of tumors 32. Thus, these findings suggest that BRD7 functions as a tumor suppressor in breast cancer in p53 dependent and independent manners.

In this study, we investigated the underlying mechanism how BRD7 functions as a tumor suppressor in breast cancer in p53 dependent manner. Our findings showed that BRD7 significantly increases p53 protein levels and changes its targeted genes at both mRNA and protein levels via activating transcriptional activity of p53. However, BRD7 overexpression did not alter the p53 mRNA levels, suggesting that BRD7 regulates p53 expression through post-translational modifications. Through chromatin immunoprecipitation coupled with high-throughput sequencing (ChIP-seq), hundreds of target genes of BRD7 have been identified, and most of them are involved in the cell cycle and apoptosis pathways, including p53 33. P53 protein is unstable with a very short half-life in cells that is degraded by ubiquitin-proteasome system. Although previous study has found that BRD7 interacts with p53 and p300 and is recruited to target gene promoters, affecting histone acetylation, p53 acetylation and promoter activity 15. In addition, BRD7 joined the polycomb repressive complex 2 (PRC2), the nucleosome remodeling and histone deacetylation (NuRD) complex at the damaged DNA and recruits E3 ubiquitin ligase RNF168 to the DNA double-strand breaks 34. However, whether BRD7 regulates the stability of p53 remains unknown. Notably, we here found that BRD7 increased the half-life and reduced ubiquitination of p53 protein.

In addition to regulation in acetylation, comprehensive cross-family structural analysis for bromodomains reveals a strong influence of flanking posttranslational modifications, such as phosphorylation, suggesting that BRDs recognize combinations of marks rather than singly acetylated sequences 35. Our findings further revealed that overexpression of BRD7 inhibited the phosphorylation of AKT, and subsequent decreased phosphorylation of MDM2, but did not alter the levels of total MDM2. Activate Akt kinase phosphorylates MDM2, and the activated MDM2 binds to p53 to initiate the ubiquitin-proteasome-mediated degradation pathways 36-38. MDM2 mainly regulates the function of p53 through three mechanisms: (1) After combined with p53 in the nucleus, MDM2 directly ubiquitinates p53 through its E3 ubiquitin ligase activity to promote the proteasome degradation; (2) the interaction between MDM2 and p53 hinders the binding of p53 to its target DNA, making p53 an ineffective transcription factor; (3) MDM2 promotes the export of p53 from the nucleus, so that p53 cannot bind to the target DNA, and further reduces its transcriptional regulation ability 39. We found that BRD7 could decreased the binding of phosphorylated MDM2 on p53, which may result in accumulation of p53 protein and activation of the downstream target genes. However, we could not exclude that the directly binding of BRD7 with p53 may be another potential mechanism of BRD7 decreasing the binding of p53 with p-MDM2 to regulate p53 ubiquitin and protein stabilization, which need to be further studied in our future work.

Moreover, we revealed that BRD7 decreased the interaction of phosphorylated MDM2 with p53 depending on its bromodomain. BRD7 binds acetylated histones via its bromodomain, and helps to sustain a proper acetylation status of histones surrounding p53-binding sites 15. In addition to recognizing acetylation motifs, bromodomain proteins also participate to ubiquitination process. For example, BRD4 recognized acetylated lysine 146 (K146) and K187 on Snail to exclude Snail recognition by its E3 ubiquitin ligases FBXL14 and β-Trcp1, thereby inhibiting Snail polyubiquitination and proteasomal degradation 40. The BRD of CREB-binding protein (CBP) interact with small ubiquitin-like modifier 1 (SUMO-1), and function as an intramolecular E3 ligase for SUMOylation of the cell cycle regulatory domain 1 (CRD1) of CBP 41. The transcriptional intermediary factor 1 (TIF1) γ's ability to ubiquitinate its substrate Smad4 requires its PHD finger-bromodomain, as does its transcriptional repressor activity 42. Bromodomain and WD repeat domain containing 3 (Brwd3) functions as a Damage-specific DNA binding protein 1- and CULLIN (CUL)4-associated factor in a Cullin4-RING Finger E3 Ligase, inducing ubiquitylation of *Drosophila* cryptochrome and its light induced degradation 43. It seems conceivable that BRD7 can regulate the interaction of phosphorylation of MDM2 through acetylating p53 via its bromodomain 44, 45.

Furthermore, we also demonstrate that silencing of p53 by RNAi or deletion of bromodomain partly rescues the inhibitory effects of BRD7 *in vitro* and *in vivo*, supporting that BRD7 functions as tumor suppressor not only dependent p53 pathway, but also other signaling involved in cell-cycle arrest and apoptosis induction. In other hand, BRD7 binds to the inter-SH2 (iSH2) domain of p85 through an evolutionarily conserved region located at the C terminus of BRD7, and this interaction can lead to an increase in p110 proteins and in PI3K pathway signaling 26. And the amino terminal part of BRD7 upstream of its bromodomain interacts with p53 to function its inhibitory effects on tumor cells growth 15. Thus, the mutant type of BRD7 lacking the bromodomain still retains a part of suppressive functions.

## Conclusion

We identify BRD7 as a positive regulator for wild type p53, but not mutant p53. BRD7 decreases the binding of phosphorylated MDM2 on p53 through decreasing AKT phosphorylation. Therefore, BRD7 functions as a tumor suppressor by stabilizing p53 level in breast cancer, which gives clinical significances in gene-therapeutics.

## Supplementary Material

Supplementary figure.
