# Supplementary material for: BRD7 Stabilizes P53 via Dephosphorylation of MDM2 to Inhibit Tumor Growth in Breast Cancer Harboring Wild-type P53
Source: J Cancer. 2022 Feb 28;13(5):1436–48. doi: 10.7150/jca.67447 (PMC8965117; doi:10.7150/jca.67447)
Supplement: Supplementary file 1 — Supplementary figure. [file jcav13p1436s1.pdf]

Supplementary Figure 1

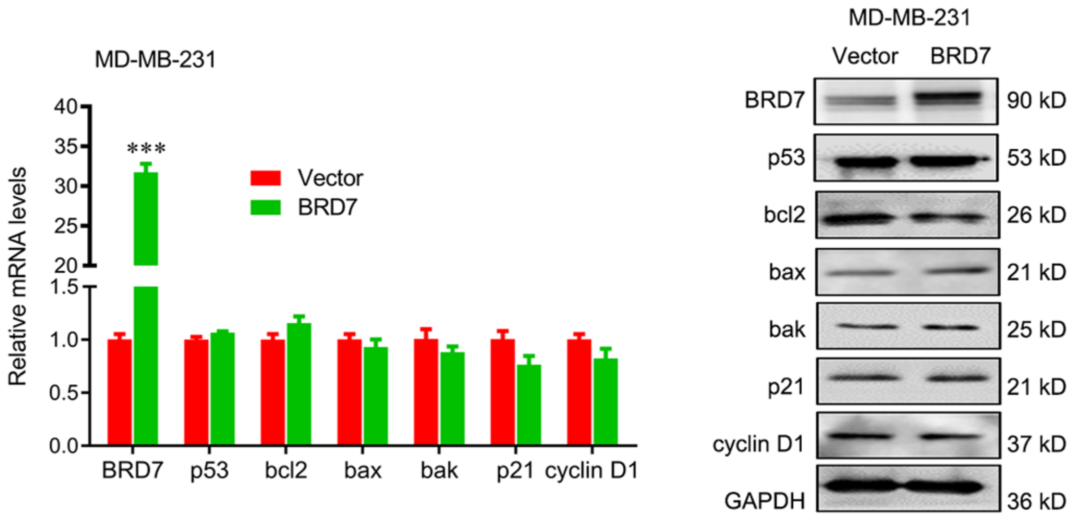

Figure S1 BRD7 cannot regulate mutant p53 protein expression and its downstream signaling. (A) Western blot analysis for p53 and its target genes, Bcl-2, Bax, Bak, p21 and cyclin D1 in MD-MB-231 cells transfected with plasmid vector or BRD7 expressed plasmid. ***p < 0.001 vs vector. (B) Relative mRNA levels of p53 and its target genes, Bcl-2, Bax and Bak, in MDA-MB-231 cells transfected with vector plasmid or BRD7 expressed plasmid measured by real-time PCR.
